# Sexual dimorphic function of IL-17 in salivary gland dysfunction of the C57BL/6.NOD-*Aec1Aec2* model of Sjögren’s syndrome

**DOI:** 10.1038/srep38717

**Published:** 2016-12-13

**Authors:** Alexandria Voigt, Lida Esfandiary, Arun Wanchoo, Patricia Glenton, Amy Donate, William F. Craft, Serena L. M. Craft, Cuong Q. Nguyen

**Affiliations:** 1 Department of Infectious Diseases and Pathology, University of Florida College of Veterinary Medicine, 2015 SW 16^th^ Ave, Gainesville, Florida 32611, USA; 2 Center for Orphan Autoimmune Disorders, University of Florida College of Dentistry, 1600 SW Archer Rd, Gainesville, Florida 32610, USA

## Abstract

Interleukin (IL)-17 is one of the critical inflammatory cytokines that plays a direct role in development of Sjögren’s syndrome (SjS), a systemic autoimmune disease characterized by a progressive chronic attack against the exocrine glands. The expression levels of IL-17 are correlated with a number of essential clinical parameters such as focus score and disease duration in human patients. Significantly immunological differences of Th17 cells were detected at the onset of clinical disease in female SjS mice compared to males. To further define the role of IL-17 in SjS and elucidate its involvement in the sexual dimorphism, we examined the systemic effect of IL-17 by genetically ablating *Il-17* in the C57BL/6.NOD-*Aec1Aec2*, spontaneous SjS murine model. The results indicate that IL-17 is a potent inflammatory molecule in the induction of chemoattractants, cytokines, and glandular apoptosis in males and females. Elimination of IL-17 reduced sialadenitis more drastically in females than males. IL-17 is highly involved in modulating Th2 cytokines and altering autoantibody profiles which has a greater impact on changing plasma cells and germinal center B cell populations in females than males. The result supports a much more important role for IL-17 and demonstrates the sexual dimorphic function of IL-17 in SjS.

Sjögren’s syndrome (SjS) is a complex chronic autoimmune disease that targets the exocrine glands, predominantly the salivary and lacrimal glands, which results in xerostomia and keratoconjunctivitis sicca. While SjS can be diagnosed as an independent disease, referred to as primary SjS (pSjS), it is often seen in association with other connective tissue disease, referred to as secondary SjS^1^,^2^. The underlying pathogenesis of SjS is still elusive, but it is thought to involve abnormal salivary gland homeostasis, neural circuitry malfunction from the presence of autoantibodies, and progressive tissue destruction mediated by infiltrating lymphocytes^2^. The multi-factorial etiology further complicates the systemic manifestations of the disease. In particular, the gastrointestinal tract, skin, lungs, vasculature, kidneys, bladder, and vagina are among those organs afflicted. Involvement of the musculature often leads to fibromyalgia-like symptoms and chronic fatigue. Between 4% and 10% of patients with SjS will develop non-Hodgkin’s B cell lymphomas, some of which become high-grade malignancies^3^,^4^.

One of the hallmark pathological changes is the presence of lymphocytic foci (LF) in the exocrine glands^5^. These foci are composed mainly of B and CD4^+^ T cells with sporadic numbers of macrophage and dendritic cells (DC). About 25% of patients develop foci that resemble germinal center (GC) structure^6^. Reksten *et al*.^7^ demonstrated that GC^+^ patients had significantly increased levels of IL-1RA, IL-15, IL-17A (IL-17) and IFN-α, and of chemokines including macrophage inflammatory proteins (MIPs)-1α, MIP-1β, eotaxin and MCP-1, with higher levels of autoantibodies against Ro-52, Ro-60 and La-48 compared with GC^−^ patients. We and others have indicated a drastic increase in the number of T helper (Th)-17 cells and IL-17 in the glands of humans and animal models of SjS^8^,^9^,^10^,^11^. In addition, we have shown that retrograde cannulation of adenovirus serotype 5 (Ad5) vectors expressing IL-17 were able to induce a SjS-like disease profile, including the appearance of lymphocytic foci, increased cytokine levels, changes in antinuclear antibody profiles, and temporal loss of saliva flow^12^. In contrast, blocking IL-17A in spontaneous SjS-susceptible (SjS^s^) C57BL/6.NOD-*Aec1Aec2* mice reduced the disease pathology significantly^13^. Further study by Lin *et al*.^14^ clearly demonstrated that C57BL/6 mice immunized with salivary gland proteins developed overt SjS symptoms with increased Th17 cells detected in LF of the glands, however, immunized IL-17 knockout (KO) mice lacked SjS induction. An increasing amount of evidence of elevated serum and glandular Th17 cells in human patients points to the significant role of Th17 in the development of SjS^8^,^10^,^15^. Our recent study has indicated that Th17 cells, and not Th1 and Th2 cells, are highly upregulated in female SjS^s^ mice relative to male SjS^s^ mice. Remarkably, SjS^s^ mice exhibit a strong sexual dimorphism in which there is an earlier onset of sialadenitis in males, whereas female SjS^s^ mice showed a delayed, but more rapid secretory loss than males. This coincided with a higher composition of infiltrating B and T cells in the salivary glands, which exhibited stronger proliferative potential in females. These observations support one of the most remarkable aspects of SjS – the highest sexual dimorphism of rheumatic diseases with females affected 10–20 times more frequently than males^16^,^17^.

These findings warrant the question as to what is the underlying mechanism(s) that mediates the induction of SjS by IL-17 or Th17 cells, and how the disease process is modulated by IL-17 differently in males and females. There is evidence to suggest that secretion of IL-17 by salivary gland epithelial cells leads to sequestration of neutrophils and monocytes in the glands^18^,^19^,^20^. The initial sequestration of inflammatory cells is thought to be mediated by some sort of glandular perturbation, e.g. abnormal gland development, aberrant patho-physiological changes, unregulated apoptosis, or atrophic gland function^21^. The recruitment of innate immune cells facilitates an influx of autoantigen-specific B and T cells. In recent studies, the majority of the antigen-specific T cells are Th17 cells reactive against the type III muscarinic receptor (M3R)^22^,^23^. The experienced Th17 cells are capable of inducing gland destruction as well as promoting the growth and maturation of autoreactive B cells. Autoantibodies produced by the expansion of selective B cells can instigate glandular dysfunction leading to the shutdown of saliva flow. Recent evidence has indicated that estrogen is capable of inducing Th17 cells by activation of its Rorγt transcription factor^24^. Furthermore, estrogen can also promote IL-4-producing Th2 cells resulting in the activation of B cells to produce high levels of immunoglobulins and autoantibodies^25^. The role of androgen on the immune response is still controversial but generally it is thought to exert suppressive potential^26^,^27^. Studies have indicated that, like estrogen, androgen has an important role in preventing apoptosis of gland epithelium and in SjS glands^28^,^29^. When androgen regulation is defective, acinar atrophy and ductal cell hyperplasia can occur^30^.

Our data indicate that IL-17 is responsible for the clinical signs of SjS^12^,^13^, however its role in glandular apoptosis, B cell function, and sexual dimorphism has not been examined. In the present study, we sought to test the hypothesis that Th17 cells are unique antigen-specific T cells responsible for the development of autoantibodies, which target the destruction of the salivary glands and explore whether a sex difference in IL-17 exists in a spontaneous SjS^s^ B6.NOD-*Aec1Aec2* animal model. Our results demonstrate that the elimination of IL-17 restored gland secretory function and reduced sialadenitis more drastically in females. Our data indicate that IL-17 is a potent inflammatory molecule in the induction of chemoattractants, cytokines, and glandular apoptosis in males and females. Analysis of the B cell response revealed that IL-17 is highly involved in modulating Th2 cytokines and altering autoantibody profiles while changing plasma cell and germinal center B cell populations in female and male SjS^s^ mice. The study demonstrates a pivotal role of IL-17 and Th17 cells in the pathogenesis of SjS, and more importantly that IL-17 exhibits a sex difference in the disease process.

## Results

### IL-17 is directly involved in salivary gland function of male and female B6.NOD-*Aec1Aec2* mice

**Figure 1:**
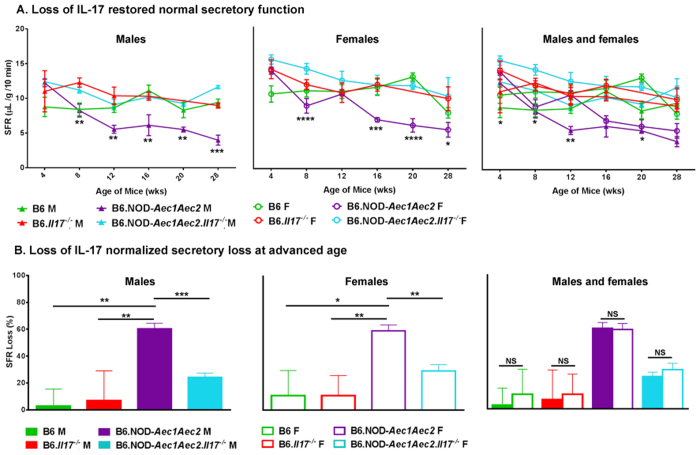
Loss of IL-17 restores normal secretory function of the salivary glands. Stimulated saliva were collected and measured. (**A**) Restored normal saliva flow of male and female mice. The saliva flow rate for males, females and males with females (SFR ± SEM, presented as μL/gram/10 min) is shown from 4–28 weeks of age (B6 F, n = 8; B6 M, n = 7; B6.*Il17*^*−/−*^ F, n = 5; B6.*Il17*^*−/−*^ M, n = 5; B6.NOD-*Aec1Aec2* F, n = 7; B6.NOD-*Aec1Aec2* M, n = 6; B6.NOD-*Aec1Aec2.Il17*^*−/−*^ F, n = 5; B6.NOD-*Aec1Aec2.Il17*^*−/−*^ M, n = 10) for males. The statistical differences were determined using one-way ANOVA where **p < 0.005, ***p < 0.001 and ****p < 0.0001. (**B**) Normal saliva flow at advanced age in males and females. The percentage of the loss of SFR between the 4-week and 28-week time points is displayed ± SEM for males, females and males with females. The statistical differences were determined using one-tailed unpaired t-test with Welch’s correction (NS: not significant, *p < 0.05, **p < 0.01, and ***p < 0.001).

IL-17 levels are highly elevated in the salivary glands of human with SjS and animal model of SjS^8^. The upregulation of IL-17 has been shown to correlate with the severity of the disease^15^, while short-term blockage of IL-17 in the glands using gene therapy has improved some of the clinical signs in spontaneous SjS^s^-B6.NOD-*Aec1Aec2* mice^13^. Recent data have indicated that Th17 cells are critical in the development of SjS in an immunized SjS model with female mice^14^. To understand the role of IL-17 in gland function and to determine if IL-17 has an effect on secretory function, we genetically eliminated the *Il17* gene in the B6.NOD-*Aec1Aec2* mice. Our data indicated that B6.NOD-*Aec1Aec2.Il17*^*−/−*^ mice appeared to have improved glandular function and restored normal saliva flow rate (SFR) from 4–28 weeks of age in both males and females (Fig. 1A). B6 and B6.*Il17*^*−/−*^ mice showed no significant loss of saliva from 4–28 weeks of age. As expected, B6.NOD-*Aec1Aec2* mice exhibited significant loss of SFR over similar age span. The decrease in SFR with advanced age (28 weeks) was more than 80% of normal baseline at 4 weeks of age in male and female B6.NOD-*Aec1Aec2* mice, and the loss was significantly reduced in both sexes of IL-17KO SjS^s^ mice (Fig. 1B). The result provides a clear indication that IL-17 has similar effects on salivary secretory function in males and females.

### Loss of IL-17 exhibited more significant improvement of sialadenitis in females than males

**Figure 2:**
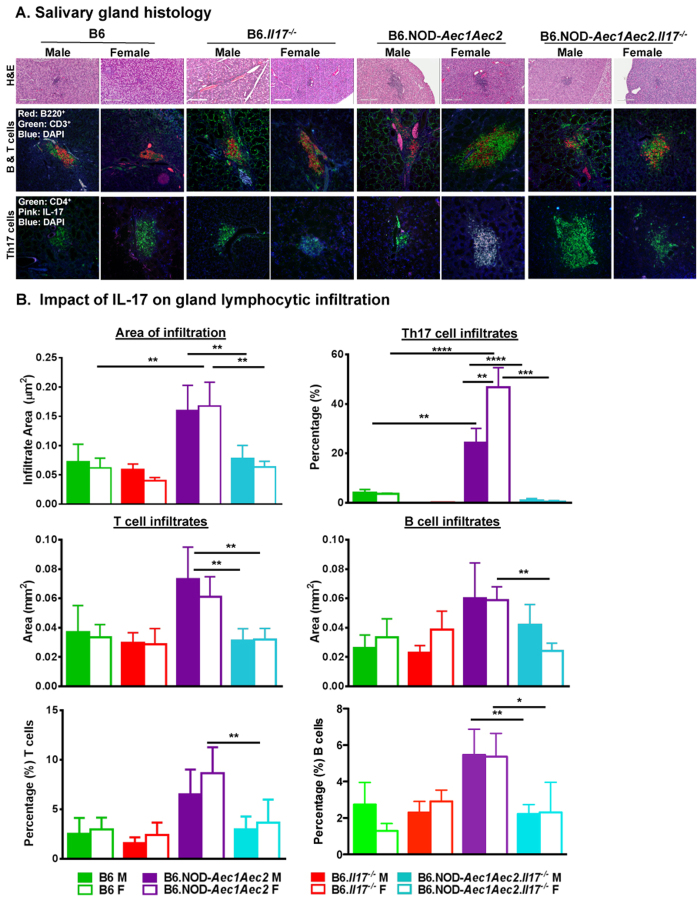
The effect of IL-17 on lymphocytic infiltrates in the salivary glands. (**A**) Histological examination of the salivary glands. Glands were excised from all lines at 30 ± 4 weeks of age. Paraffin-embedded glands were sectioned and stained for H&E (B6 F, n = 5; B6 M, n = 5; B6.*Il17*^*−/−*^ F, n = 5; B6.*Il17*^*−/−*^ M, n = 5; B6.NOD-*Aec1Aec2* F, n = 6; B6.NOD-*Aec1Aec2* M, n = 3; B6.NOD-*Aec1Aec2.Il17*^*−/−*^ F, n = 5; B6.NOD-*Aec1Aec2.Il17*^*−/−*^ M, n = 5). Glands were stained for B220^+^B cells (red), CD3^+^ T cells (green), and CD4^+^ (green) and IL-17^+^ (pink) for Th17 with DAPI nuclei staining (blue). Negative controls were performed with rabbit IgG isotype. All representative images were presented at 200x magnification. (**B**) Reduction in lymphocytic infiltrates in males and females. Total area of infiltration, B cells, T cells, and Th17 cells in the salivary glands were determined using densitometrical analysis with ROI and/or threshold setting using Nikon Elements Software. Percentage of B and T cells was determined by flow cytometric analysis gated on live B220^+^B cells and CD3^+^ T cells. The statistical significance was calculated by one-tailed Mann-Whitney tests where error bars indicate SEM *p < 0.05, **p < 0.01, ***p < 0.001 and ****p < 0.0001.

Previous studies have demonstrated that aberrant pathophysiological changes are detected in 4 week old B6.NOD-*Aec1Aec2* mice, followed by lymphocytic infiltration at approximately 16–20 weeks of age in male and female B6.NOD-*Aec1Aec2* mice, preceded by the loss of secretory function in both sexes^31^,^32^. Our recent study showed that severe sialadenitis occurs earlier in female SjS^s^ mice than the male counterpart during the adaptive phase with progressive severity during the clinical-disease phase. In order to compare the inflammatory lesions in the salivary glands, we used male and female B6.NOD-*Aec1Aec2* mice with sex- and closely age-matched B6 controls to determine the degree of sialadenitis in the glands at the end stage of the disease. Enumerating the focus score, the results indicated that male and female diseased B6.NOD-*Aec1Aec2* mice exhibited higher focus scores (1.250 ± 0.313, 1.619 ± 0.381, respectively) in comparison to male and female B6 (0.400 ± 0.163, 0.385 ± 0.140) or B6.*Il17*^*−/−*^ (0.500 ± 0.211,0.333 ± 0.500) mice. Interestingly, female SjS^s^ mice deficient in *Il17* showed more than a 2-fold decrease in focus scores, while male SjS^s^ mice deficient in *Il17* showed a slight decrease in focus scores compared to male or female SjS^s^ mice (Table 1, Fig. 2A). As demonstrated in Fig. 2B, evaluating the overall area of sialadenitis showed that glands of both sexes of SjS^s^ mice were heavily infiltrated at similar levels with higher levels of Th17 cells in females than in males. Ablation of *Il-17* significantly reduced the area of sialadenitis, CD3^+^ T cells and Th17 cells, in both sexes of SjS^s^ mice. Detailed examination of the lymphocytic infiltrate revealed that in addition to a lower focus score, male and female B6.NOD-*Aec1Aec2.Il17*^*−/−*^ mice showed smaller areas of infiltration with a significant decrease in the area of CD3^+^ T cells and CD4^+^IL-17^+^ Th17 cells compared to both sexes of B6.NOD-*Aec1Aec2* mice. Additionally, B cell levels were reduced in female B6.NOD-*Aec1Aec2.Il17*^*−/−*^ mice. Histological evaluation revealed that female B6.NOD-*Aec1Aec2* displayed higher numbers of neutrophils and macrophages than their male counterparts whereas minimal numbers of neutrophils and macrophages were detected in both sexes of B6 and B6. *Il17*^*−/−*^ mice. Interestingly, male and female B6.NOD-*Aec1Aec2.Il17*^*−/−*^ mice showed smaller aggregates of inflammatory cells and an absence of neutrophils and macrophages (Supplementary Fig. S1). The results suggest that IL-17 plays an important role in influencing the numbers of inflammatory cells as well as the cell type in inflammatory infiltrate organization. Eliminating *Il17* led to a more pronounced change in focus score in females than males, and with similar areas of total infiltrates, lower frequency of B and T cells and a significant reduction of neutrophil and macrophage infiltration.

### Loss of IL-17 reduces the pro-inflammatory response and mitigates glandular apoptosis predominantly in female SjS^s^ mice

**Figure 3:**
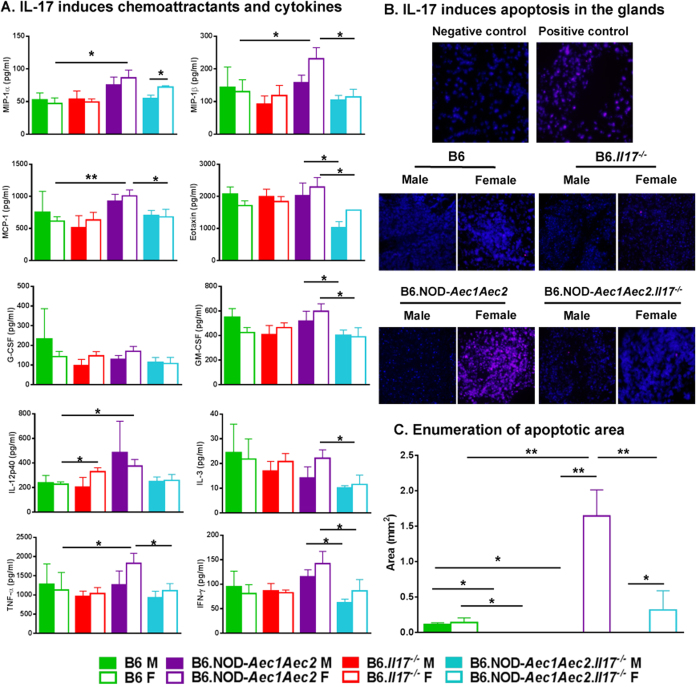
Loss of IL-17 reduces chemoattractants and proinflammatory cytokines with diminished apoptosis. (**A**) Decrease in specific chemoattractants and cytokines in males and females. Sera of four mouse strains (B6 F, n = 5; B6 M, n = 5; B6.*Il17*^*−/−*^ F, n = 3; B6.*Il17*^*−/−*^ M, n = 5; B6.NOD-*Aec1Aec2* F, n = 14; B6.NOD-*Aec1Aec2* M, n = 4; B6.NOD-*Aec1Aec2.Il17*^*−/−*^ F, n = 5; B6.NOD-*Aec1Aec2.Il17*^*−/−*^ M, n = 5) were collected at 30 ± 4 weeks of age. The levels of chemokines and cytokines were determined using mouse Biorad Bio-Plex cytokine kit. The statistical significance was calculated by one-tailed Mann-Whitney tests where error bars indicate SEM with NS: not significant, *p < 0.05, **p < 0.01. (**B**) The pronounced effect of IL-17 in females during the late stage of apoptosis. Apoptosis was determined using TUNEL assay in which salivary glands were digested with Proteinase K, incubated with TdT, biotin-16-dUTP stained with streptavidin-AF647 and DAPI nuclei staining. Negative control was not incubated with TdT and positive control was treated with DNase I prior to incubation with TdT. (B6 F, n = 5; B6 M, n = 4; B6.*Il17*^*−/−*^ F, n = 5; B6.*Il17*^*−/−*^ M, n = 5; B6.NOD-*Aec1Aec2* F, n = 9; B6.NOD-*Aec1Aec2* M, n = 5; B6.NOD-*Aec1Aec2.Il17*^*−/−*^ F, n = 3; B6.NOD-*Aec1Aec2.Il17*^*−/−*^ M, n = 4). (**C**) Area of apoptosis is quantified using Nikon Element software. The statistical significance was calculated using one-tailed Mann-Whitney tests where error bars indicate SEM *p < 0.05, **p < 0.01. Representative images were presented at 400x magnification.

Minor salivary glands (MSG) of human patients are affected with infiltrates composed mainly of CD4^+^ T and B cells, similar to the mouse models of SjS. Macrophages (MΦs), neutrophils and dendritic cells (DCs) of MSG made up a small, but considerable, number of infiltrating mononuclear cells (MNC) with few natural killer cells (NKs). The influx of lymphocytes is mediated by the activation of adhesion molecules and chemokines like MIP-1α, MIP-1β, RANTES, IL-8, and CCL20^33^,^34^. It is not evident if inflammatory lesions precede or occur secondary to gland apoptosis, however Fas, FasL, and apoptosis can be observed in glands of SjS humans and mice^35^,^36^. As previously demonstrated, the loss of IL-17 impeded the influx of lymphocytes, with a more pronounced reduction of lymphocytes in female than male SjS^s^ mice. To understand the systemic influence of IL-17 on other essential cytokines, and to further explore the contribution of IL-17 to gland apoptosis in both sexes, we examined a number of chemoattractants and inflammatory cytokines and their role in apoptosis. As presented in Fig. 3A, chemoattractant proteins like MCP-1 for monocytes, eotaxin for eosinophils as well as MIP-1α and MIP-1β levels were highly upregulated in both sexes of SjS^s^ mice. The loss of IL-17 in the B6.NOD-*Aec1Aec2.Il17*^*−/−*^ mice showed a drastic decrease in MIP-1β, MCP-1, and eotaxin, in which levels were significantly reduced in all three chemoattractants in females, whereas only eotaxin was significantly reduced in males. The decline in the chemoattractants was also coincided with the decrease of other cytokines, specifically GM-CSF, IL-3, TNF-α, and IFN-γ in female SjS^s^ mice and only GM-CSF and IFN-γ in male SjS^s^ mice. We and others have shown that IFN-γ and TNF-α can mediate epithelial cell apoptosis^37^,^38^,^39^. Therefore, we sought to determine whether a decrease in these inflammatory cytokines by knocking out IL-17 would have an effect on gland cell death. TUNEL staining indicated that female B6.NOD-*Aec1Aec2* mice exhibited a marked increase in apoptotic cells, while male mice exhibited minimal cell death. Ablation of IL-17 showed significantly decreased apoptotic cells in female SjS^s^ mice. Male and female B6 mice displayed similar basal levels of apoptotic cells, and ablation of IL-17 effectively improve glandular apoptosis in both sexes (Fig. 3B,C). Similar results were observed when examined for cleaved Caspase-3 in the salivary glands (Supplementary Fig. S2). Our data indicate that IL-17 plays a significant role in increasing chemoattractants with a stronger effect in female SjS^s^ mice, therefore, eliminating it reduces pro-inflammatory cytokines and lessens gland apoptosis in females and exerts a moderate effect on male SjS^s^ mice.

### IL-17 alters autoantibody production and response by affecting germinal center B cells and plasma cells

**Figure 4:**
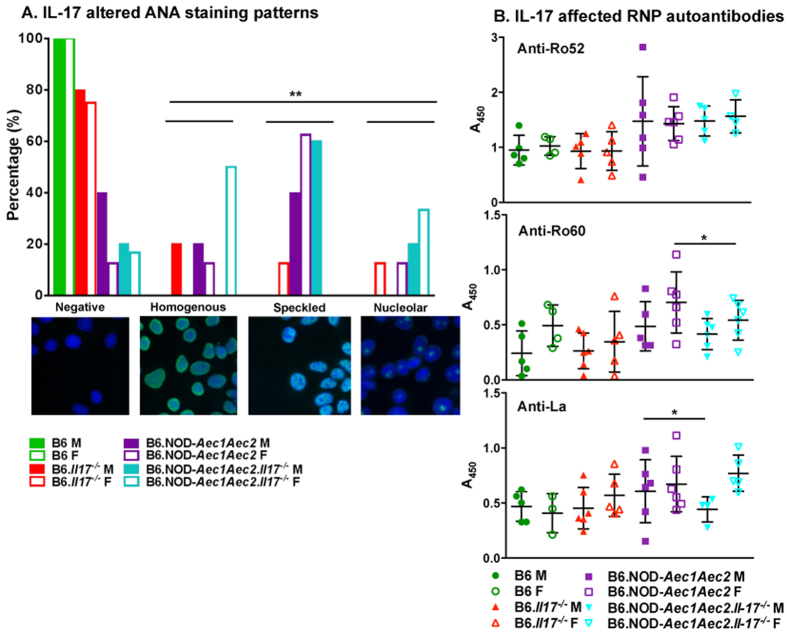
The sexual dimorphic effect of IL-17 on anti-nuclear autoantibody response. (**A**) Higher percentage of speckled ANA pattern in males and homogenous and nucleolar patterns in females. Sera of mice (B6 F, n = 5; B6 M, n = 5; B6.*Il17*^*−/−*^ F, n = 8; B6.*Il17*^*−/−*^ M, n = 5; B6.NOD-*Aec1Aec2* F, n = 8; B6.NOD-*Aec1Aec2* M, n = 5; B6.NOD-*Aec1Aec2.Il17*^*−/−*^ F, n = 6; B6.NOD-*Aec1Aec2.Il17*^*−/−*^ M, n = 5) were incubated with HEp2 cells, followed by AF488 goat anti-mouse IgG (green) and DAPI (blue). Representative images of negative, homogenous, nucleolar, and speckled staining patterns are given at 100x magnification. The bar graph displays the frequency of occurrence of each of the different staining patterns. The experiment was repeated twice. A Chi squared test was performed, where **p < 0.001. (**B**) Decrease in anti-La in males and anti-Ro60 in females. Changes in anti-Ro52, anti-Ro60, and anti-La autoantibodies in males versus females by IL-17 were determined by ELISA. The statistical significance was calculated using one-tailed Mann-Whitney tests, *p < 0.05.

In addition to apoptosis as one of the etiologies of SjS, accumulating evidence suggests that disturbances in B lymphocyte homeostasis, including their role in ectopic germinal center formation in lacrimal and salivary gland tissue, are major features of SjS^40^. B cell hyper-reactivity is clearly documented in SjS, leading to hypergammaglobulinemia, production of an expanding set of autoreactive antibodies, and at times the monoclonal expansion of B cells capable of transformation to B-cell lymphomas in a subset of patients. Although antibody production is not present in all patients, it remains a valid diagnostic criterion. To examine whether IL-17 was capable of affecting the autoantibody profile in the SjS^s^ mice, we stained Hep2 cells for ANA. As illustrated in Fig. 4A, more than 60% of female SjS^s^ mice exhibited speckled pattern with 40% of males showing speckled or no staining pattern, and both sexes of B6 mice were negative. Ablation of *Il-17* in B6.NOD-*Aec1Aec2* mice indicated that there is a significant shift in ANA pattern from predominantly speckled to more homogenous and nucleolar staining patterns in which 50% of female showed homogenous and 30% with nucleolar pattern, while male B6.NOD-*Aec1Aec2.Il17*^*−/−*^ mice increased to 60% with speckled staining with 20% positive for nucleolar or negative. Examining individual RNP autoantibodies indicated that male and female SjS^s^ mice exhibited high levels of anti-Ro52 compared to B6 or B6.*Il17*^*−/−*^ mice and showed minimal changes with *Il17* ablation. However, ablating *Il17* appeared to reduce more anti-Ro60 in female than male SjS^s^ mice, while the trend of anti-La was reversed in which male SjS^s^ mice were more negatively affected by *Il17* ablation than females (Fig. 4B).

**Figure 5:**
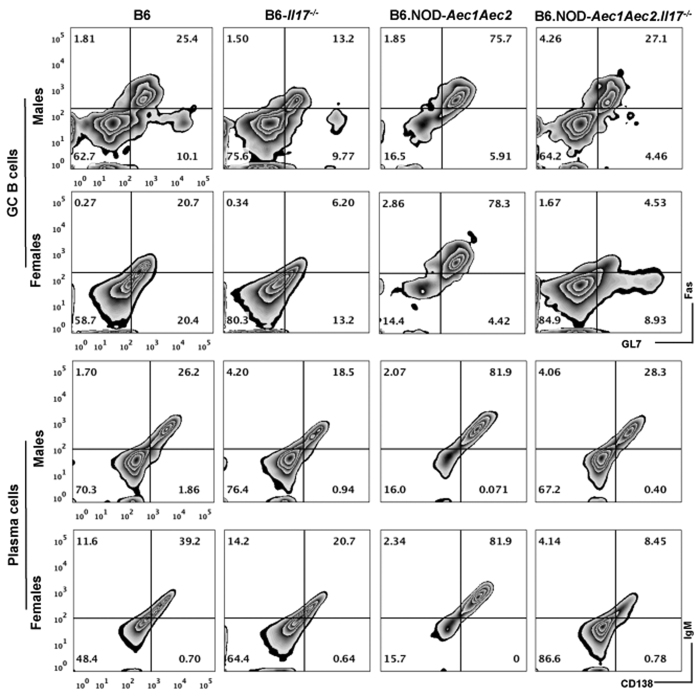
Sexual dimorphic effect of IL-17 on germinal center B cells and plasma cells. More significant loss of germinal center (GC) B cells and plasma cells in salivary glands of females than males. Single-cell suspension was obtained from the salivary glands of male and female B6, B6.*Il17*^*−/−*^, B6.NOD-*Aec1Aec2*, and B6.NOD-*Aec1Aec2.Il17*^*−/−*^ (n = 3/strain/sex) and stained with live/dead marker and anti-CD19-AF647, anti-Fas-PE, anti-GL7-FITC, anti-B220-PE Cy7, anti-CD138-BV605 and anti-IgM-Pacific Blue. Live cells positive for B220 were analyzed for CD138^+^IgM^+^ to identify plasma cells and germinal center B cells were identified as live CD19^+^GL7^+^Fas^+^ cells. The data were acquired by BD Fortessa and analyzed by FlowJo (Ashland, OR). Representative images from each group are presented.

The shifting in the ANA patterns and anti-RNP could be the result of the changing germinal center B cell and plasma cell numbers as presented in Fig. 5 and Table 2. Examination of the salivary glands revealed a 58% and 24% decrease in GC B and plasma cells, respectively in male and 24% and 46% in female B6*.Il17*^*−/−*^ mice. Male and female SjS^s^ mice showed elevated percentages of GC B and plasma cells. Interestingly, ablation of IL-17 in SjS^s^ mice showed 58% and 59% reduction of GC B and plasma cells in males respectively, however in female SjS^s^ mice, deficiency of IL-17 led to 87% and 80% reduction of GC B and plasma cells. These results clearly indicate a stronger effect on GC B and plasma cells in females than males in the context of SjS.

**Figure 6:**
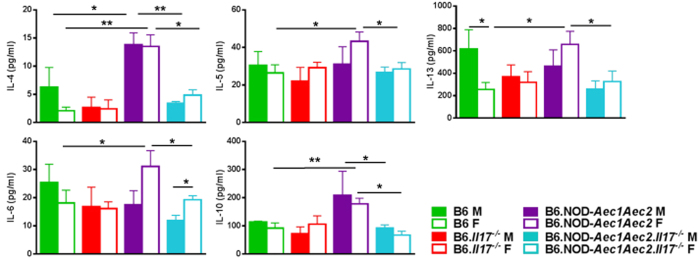
Sexual dimorphic effect of IL-17 on sera Th2 cytokines. IL-5, IL-13, and IL-6 were decreased in females, and IL-4 and IL-10 were reduced in both sexes. Sera of mice (B6 F, n = 5; B6 M, n = 4; B6.*Il17*^*−/−*^ F, n = 3; B6.*Il17*^*−/−*^ M, n = 4; B6.NOD-*Aec1Aec2* F, n = 14; B6.NOD-*Aec1Aec2* M, n = 4; B6.NOD-*Aec1Aec2.Il17*^*−/−*^ F, n = 3; B6.NOD-*Aec1Aec2.Il17*^*−/−*^ M, n = 5) were analyzed using BioRad Bio-Plex manager. Graphs were generated using one-tailed Mann-Whitney t-tests in GraphPad, *p < 0.05 and ***p < 0.001.

To determine whether signature Th2 cytokines play a role in enhancing B cell survival, proliferation, and antibody production, we examined the levels of IL-4, IL-5, IL-6, IL-10, and IL-13 in the sera of males and females. As indicated in Fig. 6, female SjS^s^ mice exhibited a significant increase in these cytokines in comparison to female B6 mice, and only IL-4 was significantly elevated in male SjS^s^ compared to male B6 mice. Knocking out *Il17* in the control B6 background has little impact on the serum levels of IL-4, IL-5, IL-6, IL-10, and IL-13. However, elimination of *Il17* in B6.NOD-*Aec1Aec2* mice showed sexual dimorphic changes in serum cytokine levels, particularly IL-4, IL-5, IL-6, IL-10, and IL-13 levels were significantly reduced in female B6.NOD-*Aec1Aec2.Il17*^*−/−*^ mice, while only IL-4 and IL-10 were affected in males. These data clearly demonstrate that IL-17 affects autoantibody profile differently in males and females. The stronger sexual dimorphic impact of IL-17 on the humoral response by females is due to its more potent influence on germinal center B cells, plasma cells, and Th2-related cytokines.

## Discussion

The role of Th17 cells and its signature IL-17 cytokine have been implicated in a growing list of autoimmune diseases. Mounting evidence has suggested a significant role for IL-17 and Th17 cells in the development of SjS. In this study, we sought to determine the underlying mechanistic effect and sexual dimorphic function of IL-17 in a spontaneous animal model of SjS. Our results indicate that IL-17 is a broadly potent inflammatory cytokine, and by genetic ablation we observed significant decreases in the B and T cells, particularly Th17 cells, in the salivary glands of males and females. Our data demonstrate that female SjS^s^ mice are more strongly affected by IL-17 than males, specifically; ablation of *Il-17* in female SjS^s^ triggered marked reduction in focus score, infiltrating plasma and germinal center B cells, chemoattractants and cytokines (MIP-1β, IL-13, IFN-γ, and TNF-α), altered ANA staining patterns with a more pronounced effect on anti-Ro60, and glandular apoptosis. IL-17 plays stronger role for Th2 cytokines in females than males. Interestingly, genetic ablation of *Il-17* restores normal salivary secretion in both sexes. Collectively, our data support an essential role of IL-17 in the development of salivary dysfunction in SjS; however, our results suggest that there is a sexual dimorphic role of IL-17 in SjS.

SjS is a chronic inflammatory disease which is in part mediated by perturbation of the glandular epithelial cells resulting in the homing of monocytes and neutrophils from blood or nearby draining lymph nodes^21^,^41^. The infiltrating monocytes differentiate into macrophages and along with neutrophils, secrete inflammatory cytokines and chemokines to commence the influx of adaptive lymphocytes^42^. As a disease model, B6.NOD-*Aec1Aec2* mice produce increased levels of IL-17 that have been shown to induce the migration of neutrophils and monocytes/macrophages^8^. Our previous study indicated that female SjS^s^ mice developed an earlier onset of sialadenitis with an increased frequency and proliferative responses of Th17 cells at the onset of clinical disease compared to male mice. You *et al*. demonstrated that female SjS^s^ mice showed higher levels of IL-17A, IL-17R, IL-1α, IL-1β, and TNF-α in the conjunctiva and greater lymphocytic infiltration of the lacrimal glands and conjunctiva at 20 weeks of age compared to B6 mice^43^. In this study, female SjS^s^ expressed higher levels of serum MIP-1α, MIP-1β, and MCP-1 compared to the female wildtype in response to signals such as proinflammatory cytokines, resulting in the recruiting monocytes and neutrophils as illustrated (Fig. S1). The influx of the early innate cells induced a drastic increase in proinflammatory cytokines such as IL-12p40, TNF-α, and IFN-γ that can sustain effector cell function and glandular damage. These cytokines are in turn enhancing the positive feedback loop that leads to chronic inflammation. Genetic ablation of *Il-17* appeared to disrupt the chronic positive feedback loop and restored basal chemokine and cytokine levels, more noticeably in females than in males. A transcriptome analysis of synoviocytes stimulated with IL-17 showed that the genes significantly induced were chemokines with a chemotactic effect on neutrophils^44^. Bone marrow-derived macrophages expressed high levels of IL-17 receptors. Treatment of these cells with IL-17A induced the production of unique profiles of cytokines and chemokines, including GM-CSF, IL-3, IL-9, CCL4/MIP-1β and CCL5/RANTES^45^. Fei *et al*. have shown that TNF-α plays a central role in promoting Th17 cells and synergistically working to induce airway neutrophilia. Functionally, collaboration between TNF-α and IL-17 triggered significantly higher levels of the neutrophil chemoattractants keratinocyte cytokine and MIP-2^46^, however, these studies did not indicate the sex of the cell lines or animals, therefore it is difficult to speculate whether these differences are related to sex hormones. Estrogen can drive the differentiation of dendritic cells (DCs) and macrophages. Estrogen can also upregulate IL-17 expression, thereby eliminating IL-17 showed the more drastic effect in female SjS mice with a significant decrease in inflammatory cytokines and chemokines MIP-1β, MCP-1, IL-3, TNF-α, and IFN-γ whereas Eotaxin and GM-CSF were equally affected in both sexes.

In addition to neutrophil and monocyte recruitment, Th17 cells can function as B-cell helpers by inducing a strong proliferative response of B cells and triggering antibody production with class switching to IgG1, IgG2a, IgG2b, and IgG3, as well as the formation of germinal centers^47^. Furthermore, IL-17 cytokine signaling via CIKS (Traf3ip2) has been shown to contribute to spontaneous germinal center B cell formation and plasma cell development^48^. We observed a drastic decrease in germinal center B cells and plasma cells in salivary glands of male and female mice lacking *Il-17* in both normal and diseased backgrounds. Interestingly, female SjS^s^ mice were highly affected by the loss of *Il-17* by exhibiting a 2-fold decrease in germinal center and plasma B cells. This change in B cell populations could explain the altered autoantibody profiles. SjS^s^ mice exhibited a high frequency of homogenous/speckled ANA patterns with elevated titers for anti-SSA/Ro and anti-SSB/La^49^. Ablating *Il-17* appears to affect anti-SSA/Ro60 more so in females and anti-La in males of SjS^s^ mice. A study has indicated that B cells can produce IL-17 *in vivo* in response to infection^50^ and IL-17 has been shown to be associated with germinal center-derived autoantibodies^51^. Hormonal milieu can influence the phenotype of autoreactive B cells; for example single-cell analysis demonstrated that anti-DNA B cells in estradiol-treated mice become marginal zone cells whereas identical cells from prolactin-treated mice become follicular B cells^52^. Estrogen can also drive CD5^+^ B cells to produce autoantibodies^53^. Androgens inhibit bone marrow B lymphopoiesis^54^ and testosterone reduced the frequency of B220^+^ cells in lacrimal glands of the SjS^s^ MRL/lpr mouse model^55^. Our data indicate that IL-17 can influence the development of germinal center and plasma B cells. Further work is needed to determine the underlying mechanism of autoantibody production by these B cell populations and how sex hormones can modulate this process.

Our previous study indicated the importance of IL-4 in regulating B cell proliferation and survival during B cell ontogeny and participating in the IgM to IgG1 isotypic switch via the JAK-STAT6 signal transduction pathway^56^. SjS^s^ mice lacking *Il-4* failed to produce pathogenic IgG1 autoantibodies and showed a downregulated B cell response. As presented in this study, genetic ablation of *Il-17* reduced the IL-4 levels in both male and female SjS mice. This might be an indirect effect of IL-17. Interestingly, IL-5, IL-6, and IL-13 of other Th2 cytokines were highly affected only in female SjS^s^ mice. Females exhibit a tendency toward Th2 immune responses with increased levels of IL-4, IL-5 and IL-10. This results in the increased activity and maturation of germinal center B cells and plasma cells, thereby enhancing B cell activation. Additionally, Th17 cells have been shown to be excellent B-cell helpers. Therefore, the enhancement of Th2 response by estrogen in females is counteracted by the ablation of IL-17 positive supporting activity; therefore we see a drastic decrease in Th2 and B cell responses. The results point to the role IL-17 in regulating B cell response and function, but studies to identify the interconnected function of IL-17 directly on B cells and Th2 cytokines are ongoing.

The role of apoptosis in SjS remains controversial. Whether directly or indirectly involved in the development of secretory dysfunction in the lacrimal and salivary glands, acinar cell apoptosis is one of the histological features of SjS. It may result from glandular destruction by inflammation or by malfunction of acinar and ductal epithelial cells; apoptosis appears to affect acinar cell mass. Our data indicated that female SjS^s^ mice exhibited rampant apoptosis in the glands by TUNEL and the presence of cleaved caspases-3, whereas males showed mostly cleaved caspase-3 with minimal staining for TUNEL. These results suggest that aged female SjS mice continue to activate apoptosis signaling pathways and undergo late stage of double-stranded DNA break cell death, whereas aged males have stopped late stage of apoptosis but still maintain higher than normal apoptotic pathways. It is also possible that the male mice are appropriately clearing the late apoptotic cells, whereas there is a malfunction in either the signaling for or clearance of these cells in the female mice. Interestingly, eliminating *IL-17* showed drastic improvement in glandular cell death in both sexes, particularly the late stage of apoptosis in females. This observation could be attributed by the overall suppression of chemokines and proinflammatory cytokines resulting in broad decrease in lymphocytic infiltration, thereby minimal gland destruction was detected. Ovariectomized B6 mice displayed a significant increase in TUNEL^+^-apoptotic epithelial cells in the salivary gland cells associated with α-fodrin cleavage^57^. The level of estrogen deficiency can modulate retinoblastoma-associated protein 48 (RbAp48) to induce tissue-specific apoptosis in the exocrine glands^58^. Estrogen levels are cyclically varied and low at post menopause. It is possible the low concentration of estrogen in the aged diseased mice activate RbAp48 to induce apoptosis. The apoptosis is mediated by the proinflammatory milieu initiated by salivary epithelial cells producing IFN-γ, IL-18 and IL-17, therefore a deficiency in IL-17 appeared to counterbalance the low level of estrogen and improve gland apoptosis. Although we did not examine the lacrimal glands in this study, ablating *Il-17* in the CD25KO model of dry eye SjS created the opposite effect in the lacrimal glands in which at 16 weeks of age, deletion of *Il-17* accelerated lacrimal gland infiltration and acinar apoptosis. Deletion of *Ifn-γ* decreased caspase activity levels and TUNEL^+^ cells, whereas ablation of both *Il-17* and *Ifn-γ* ameliorated dacryoadenitis and improves glandular function^59^. Therefore, in this particular KO model, IFN-γ is more pathogenic than IL-17 in dacryoadenitis. Our previous data demonstrated that IFN-γ pathway is highly activated in the salivary glands during the early stages of the autoimmune process, whereas Th17/IL-17 pathway is activated during the later stages of the disease^8^,^22^,^60^. These observations suggest that both IFN-γ and IL-17 are essential to the development of salivary gland dysfunction, however IFN-γ is more critical in the early stage while IL-17 is more essential during the later stage.

Collectively, we have shown that IL-17 plays an essential role in the autoimmune process of SjS and the sexual dimorphism of the disease. Our data indicate that IL-17 is a potent inflammatory molecule in the induction of chemoattractants, cytokines, and glandular apoptosis in males and females. IL-17 is highly involved in modulating Th2 cytokines and altering autoantibody profiles which has a greater impact on changing plasma cells and germinal center B cell populations in female than male of SjS animal model. Ablation of *Il-17* in the spontaneous SjS^s^ mice reduced lymphocytic infiltration, diminished glandular apoptosis, and more importantly restored glandular function. The result supports a much more important role for IL-17 in the early stage and adaptive autoimmune response than previously recognized. This study demonstrates for the first time the sexual dimorphic function of IL-17 in the spontaneous murine model.

## Materials and Methods

### Animals

C57BL/6 (B6).*Il17*^*−/−*^, B6J.NOD/ShiLtJ-*Aec1Aec2* (B6.NOD-*Aec1Aec2*), B6.NOD-*Aec1Aec2.Il17*^*−/*^, and B6 mice were bred and maintained under specific pathogen free conditions in the animal facility of Animal Care Services at the University of Florida. Development and characterization of SjS-like phenotypic of the B6.NOD-*Aec1Aec2* mouse are described elsewhere^32^,^61^. Briefly, introduction of two genetic regions, one on chromosome 1 (designated *Aec2*) and the second on chromosome 3 (designated *Aec1*) derived from the NOD/LtJ mouse were bred into the B6 mouse. The B6.*Il17*^*−/−*^ strain was a generously provided by Dr. Cintia S De Paiva. It was bred with the B6.NOD-*Aec1Aec2* mouse in which subsequent litters were genotyped to confirm presence of both *Aec1* and *Aec2* as well as the knockout of *Il17* to obtain the B6.NOD-*Aec1Aec2.Il17*^*−/−*^ background. All animals were maintained on a 12 hour light-dark schedule and provided food and acidified water *ad libitum*. Mice were anesthetized with isoflurane and euthanized by cervical dislocation; their organs and tissues were freshly harvested for analyses. The University of Florida’s Institutional Animal Care approved all protocols respective to breeding and the use of animals described herein. The experimental methods were carried out in accordance with the appropriate approvals and relevant guidelines.

### Measurement of saliva flow

Individual mice were weighed and given an intraperitoneal (ip) injection of 100 μl isopreterenol (0.2 mg/1 ml of PBS) and pilocarpine (0.05 mg/1 ml of PBS) to stimulate saliva flow. Saliva was collected for 10 min from the oral cavity of individual mice using a micropipette starting one minute after injection of the secretagogue. The volume of each saliva sample was measured and saliva flow rate (SFR) was calculated as volume (μl) per gram (weight of mouse).

### Histological examination of the salivary glands

Mice were anesthetized by isoflurane and were humanely euthanized by cervical dislocation. Salivary glands and lymph nodes were immediately collected. Salivary glands were fixed in 10% phosphate-buffered formalin for 24 hours. Fixed tissues were embedded in paraffin and sectioned at a thickness of 5 μm. Deparaffinization of paraffin-embedded sections was achieved by immersion in xylene, followed by dehydration in ethanol. Hematoxylin and eosin (H&E) stained sections were observed at 200x magnification by using Nikon Eclipse Ti-E inverted microscope. A single histological section per gland per mouse was examined and lymphocytic infiltrations were defined as aggregates of >50 leukocytes. Histological examination was performed by two independent pathologists in a blinded manner.

### Immunofluorescent staining for CD3+ T cells, B220+B cells and Th17 cells in the salivary glands

Paraffin-embedded tissues of the salivary glands were sectioned and mounted onto microscope slides. Slides were pressure-cooked in Trilogy (Cell Marque, Rocklin, CA) according to manufacturer’s instructions to deparaffinize and dehydrate slides. Staining procedures for CD3^+^ T cells, B220^+^B cells and Th17 cells were followed as previously described^22^. Stained sections were visualized at 200x magnification using NikonTi-E fluorescent microscope. Nikon NIS-Elements software was use to calculate infiltrate size and composition. The infiltrate composition and size, based on the threshold intensities and backgrounds for the individual channels, was determined by Region of Interest (ROI) function as detailed previously^22^.

### Analysis of mouse cytokine levels in sera

Determining the serum cytokine levels was performed using the 23-plex-mouse cytokine group I Bioplex kit (BioRad, Hercules, CA) per manufacturer’s instruction. In brief, sera samples were diluted 1:25,000 in the provided assay buffer. A 96-well assay plate was prewet with 100 μl of assay buffer. Pre-mixed beads were added to each well in a 50 μl volume and then washed, using a magnet for bead retention, before adding 50 μl of diluted sample, standard, or blank. The assay plate was incubated for 30 minutes by shaking at room temperature in the dark. The plate was then washed prior to the addition of detection antibody. Following the detection antibody the plate was washed by adding 50 μl of Streptavidin-PE per well. The samples were in incubated for 10 minutes by shaking. The plate was washed for the final time and 125 μl of assay buffer was added. The results were acquired using BioRad Magpix. The data were analyzed using the Bio-Plex Manager software (BioRad, Hercules, CA).

### Terminal deoxynucleotidyl transferase dUTP nick end labeling (TUNEL) stain for apoptotic events

Paraffin-embedded tissues of the salivary glands were deparaffinized and dehydrated by pressure-cooking in Trilogy (Cell Marque, Rocklin, CA) according to manufacturer’s instructions. Following three 5-minute washes with PBS with Tween-20 (PBS-T), the sections were treated with proteinase K for 30 minutes. The slides were washed twice with PBS-T. The positive control was incubated with 0.5 mg DNase I (Sigma Aldrich, Saint Louis, MO) for 10 minutes. TUNEL assay was performed using Click-iT TUNEL Alexa Fluor Imaging Assays as instructed by the manufacturer (ThermoFisher, Waltham, MA). In brief, glands fixed on slides were blocked with a Tris-buffered solution and then incubated with terminal deoxynucleotidyl transferase (TdT) and Biotin-11-dUTP for 1 hour at 37 °C. The reaction was stopped with sodium citrate butter for 10 minutes of incubation. Slides were incubated for one hour with AF647 streptavidin and mounted using Vectashield DAPI-mounting medium. Stained samples were visualized at 400x magnification on Nikon Ti-E fluorescent microscope with an exposure of 200 milliseconds. Nikon NIS-Elements software was used to detect the threshold and background intensities for the individual channels, which were determined by the ROI function. The DAPI threshold signal was determined first, followed by the apoptotic signal, which yielded the area of the apoptotic cells in the entire glands. The threshold intensities were kept consistent throughout the experiment.

### Detection of antinuclear antibodies (ANA) and anti- ribonucleoproteins (RNP) antibodies in the sera

Anti-nuclear antibody detection in the sera of mice was performed with the HEp-2 ANA kit (Inova Diagnostics, Inc., San Diego, CA) as previously described^22^. Stained samples were visualized at 400x magnification on Nikon Ti-E fluorescent microscope with an exposure of 200 milliseconds. Anti-Ro52, anti-R60 and anti-La were detected using ELISA. Recombinant human Ro52/SS-A (#12700), Ro60/SS-A (#15500), and La/SS-B (#12800, Diarect, Freiburg, Germany) were used to coat individual wells of a 96-well plate at a concentration of 0.6 ug/mL in carbonate buffer pH 9.6 overnight at 4 °C. The plate was washed with phosphate buffered saline pH7.4 with 0.05% Tween 20 (PBST) and then blocked in phosphate buffered saline pH 7.4 (PBS) with 5% bovine serum albumin overnight at 4 °C. 200 uL of a 1:20 dilution of sera was added into each well, in duplicate. Sera were incubated at room temperature for 2 hours. The plate was then washed with PBST and treated with a 1:10,000 dilution of anti-mouse IgG (#SC-2005, Santa Cruz Biotech, Santa Cruz, California) conjugated to horseradish peroxidase (HRP) in PBS and incubated for 2 hours at room temperature. After washing the plate with PBST the wells were treated with 100 uL of TMB substrate solution (#00-4201-56, eBioscience, San Diego, California) for 30 minutes with shaking and then with 3N HCl to stop. The plate was read at an absorbance of 450 nm using Tecan Infinite M200 Pro spectorphotometric plate reader. The data was recorded as relative absorbance.

#### Flow cytometric analysis

Salivary glands were excised and single cells were isolated as previously described^22^. Cells were rinsed, resuspended in FACS buffer, and stained with the following: Live/Dead Fixable Aqua Dead Cell Stain Kit, for 405 emission (Life Technologies, Carlsbad, CA), PE-Cy7 rat anti-mouse B220, AF700 rat anti-mouse CD3, AF647 rat anti-mouse CD19, BV605 rat anti-mouse CD138, Pacific Blue rat anti-mouse IgM, (BD Biosciences, San Jose, CA), PE mouse anti-mouse Fas (eBioscience, San Diego, CA), FITC rat anti-mouse/human GL7 (Biolegend, San Diego, CA) for 30 minutes, on ice. Fluorescence minus one (FMO) controls were performed in which cells were stained with all fluorochrome-conjugated antibodies except the one of interest (Supplementary Fig. S3). The samples were analyzed using BD Fortessa Flow Cytometer (BD Biosciences, San Jose, CA) and analysis was performed using FlowJo VX software (FlowJo, Ashland, OR).

### Statistical analyses

Statistical evaluations were determined by using one-tailed Mann-Whitney t-tests or ANOVA analyses generated by the GraphPadInStat software (GraphPad Software, La Jolla, CA), where indicated. For the ANA staining alone, a Chi squared test was performed, also generated on the GraphPadInStat software. In all cases, p values < 0.05 were considered significant.

## Additional Information

**How to cite this article**: Voigt, A. *et al*. Sexual dimorphic function of IL-17 in salivary gland dysfunction of the C57BL/6.NOD-*Aec1Aec2* model of Sjögren’s syndrome. *Sci. Rep.* **6**, 38717; doi: 10.1038/srep38717 (2016).

**Publisher's note:** Springer Nature remains neutral with regard to jurisdictional claims in published maps and institutional affiliations.

## Supplementary Material

Supplementary Information

## Figures and Tables

**Table 1:** Focus score of the salivary glands.

Strains	Sex (n)	Mean age (weeks)	Focus score
B6	M (10)	31.00 ± 0.00^a^	0.400 ± 0.163^a^
	F (13)	30.08 ± 0.29	0.385 ± 0.140
B6.*Il17*^*−/−*^	M (4)	32.00 ± 0.00	0.500 ± 0.211
	F (6)	34.00 ± 0.00	0.333 ± 0.500
B6.NOD-*Aec1Aec2*	M (8)	32.50 ± 0.57	1.250 ± 0.313^b^
	F (21)	31.00 ± 1.54	1.619 ± 0.381^b^
B6.NOD-*Aec1Aec2.Il17*^*−/−*^	M (8)	28.00 ± 0.38	1.000 ± 0.267^c^
	F (7)	35.71 ± 2.12	0.714 ± 0.286^d^

*p < 0.5, **p < 0.01.

^a^Mean ± standard error.

^b^B6.NOD-*Aec1Aec2* males/females *vs* B6, B6.*Il17*^*−/−*^ males/female(*).

^c^B6.NOD-*Aec1Aec2* males *vs* B6.NOD-*Aec1Aec2.Il17*^*−/−*^ males (NS: not significant).

^d^B6.NOD-*Aec1Aec2* females *vs* B6 (**), B6.*Il17*^*−/−*^ (**), B6.NOD-*Aec1Aec2.Il17*^*−/−*^(*) females.

**Table 2:** GC B cells and plasma cells in salivary glands.

Strains	Sex (n)	GC B cells		Plasma cells	
		Freq. (%)	Change (%)#	Freq. (%)	Change (%)
B6	M (3)	30.60 ± 2.60^a^	58%	31.80 ± 2.80	24%
	F (3)	20.73 ± 0.26		40.03 ± 0.44	
B6.*Il17*^*−/−*^	M (3)	12.83 ± 0.37	59%	24.07 ± 5.57	46%
	F (3)	8.33 ± 1.57		21.27 ± 0.43	
B6.NOD-*Aec1Aec2*	M (3)	65.50 ± 5.10^b^	58%	72.77 ± 4.57^b^	59%
	F (3)	51.83 ± 13.31		56.40 ± 12.86	
B6.NOD-*Aec1Aec2.Il17*^*−/−*^	M (3)	27.37 ± 0.27^c^	87%	29.80 ± 1.50^c^	80%
	F (3)	6.79 ± 2.58d,^e^		11.20 ± 2.75d,^e^	

^a^Mean ± standard error.

^b^NS: Not significant (B6.NOD-*Aec1Aec2 males vs females*).

^c^B6.NOD-*Aec1Aec2* males *vs* B6.NOD-*Aec1Aec2.Il17*^*−/−*^ males, ***p < 0.001.

^d^B6.NOD-*Aec1Aec2* females *vs* B6.NOD-*Aec1Aec2.Il17*^*−/−*^ females, *p < 0.05.

^e^B6.NOD-*Aec1Aec2.Il17*^*−/−*^ males vs females, *p < 0.5.

^#^Percent decrease from B6 or B6.NOD-*Aec1Aec2* with the respective sex.
