# Supplementary material for: Sexual dimorphic function of IL-17 in salivary gland dysfunction of the C57BL/6.NOD-Aec1Aec2 model of Sjögren’s syndrome
Source: Sci Rep. 2016 Dec 13;6:38717. doi: 10.1038/srep38717 (PMC5153841; doi:10.1038/srep38717)
Supplement: Supplementary Information [file srep38717-s1.pdf]

Sexual dimorphic function of IL-17 in salivary gland dysfunction of the C57BL/6.NOD-*Aec1Aec2* model of Sjögren's syndrome

Alexandria Voigt [1], Lida Esfandiary [1], Arun Wanchoo [1], Patricia Glenton [1], Amy Donate [1], William F. Craft [1], Serena L.M. Craft [1], Cuong Q. Nguyen [1,2]

[1] Department of Infectious Diseases and Pathology, University of Florida College of Veterinary Medicine, 2015 SW 16th Ave, Gainesville, Florida 32611, USA

[2] Center for Orphan Autoimmune Disorders, University of Florida College of Dentistry, 1600 SW Archer Rd, Gainesville, Florida 32610, USA

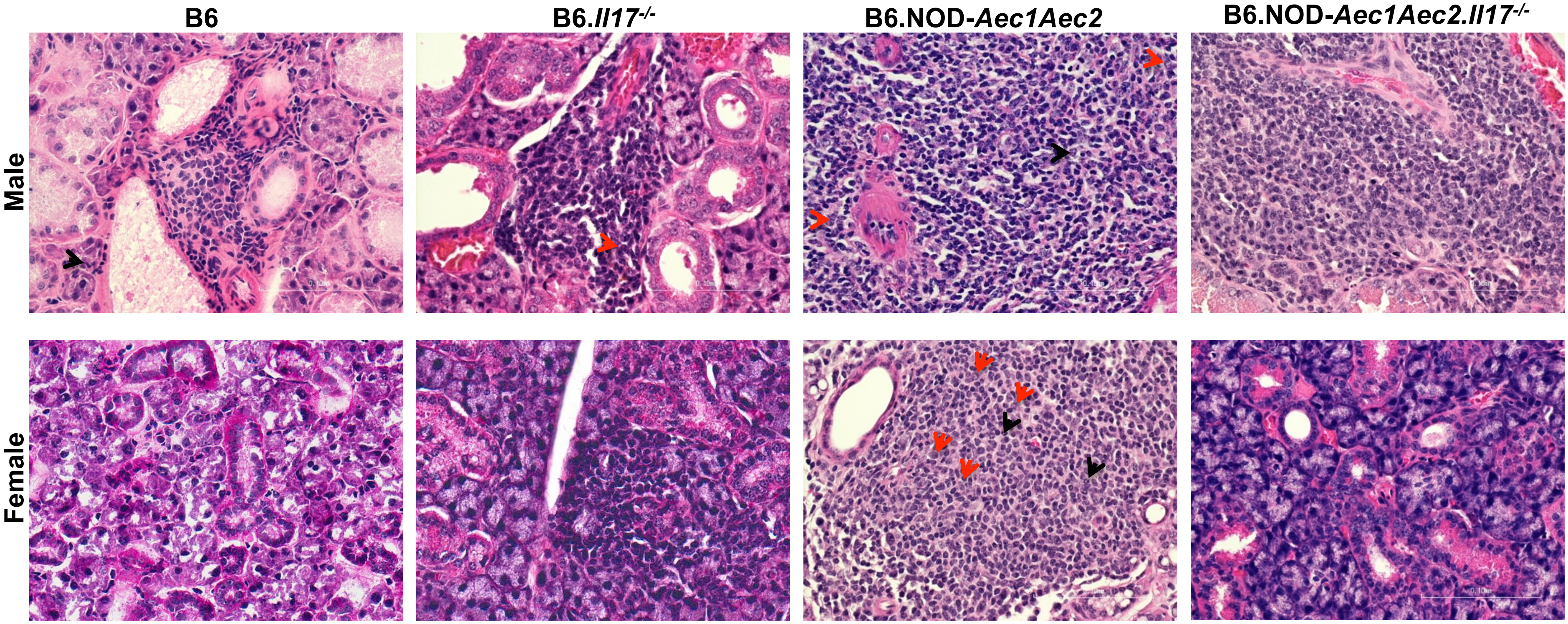
B6
B6.Il17-/-
B6.NOD-Aec1Aec2
B6.NOD-Aec1Aec2.Il17-/-
Male
Female

Supplementary Fig. S1. Histological examination for macrophages and neutrophils in the salivary glands. Salivary glands were evaluated by two veterinary pathologists who were blinded to the study results. Glands were excised from males and females of the four strains at 30 ± 4 weeks of age. Paraffin-embedded glands were sectioned and stained for H&E (B6 F, n=5; B6 M, n=5; B6.*Il17*$^{-/-}$ F, n=5; B6.*Il17*$^{-/-}$ M, n=5; B6.NOD-*Aec1Aec2* F, n=6; B6.NOD-*Aec1Aec2* M, n=3; B6.NOD-*Aec1Aec2.Il17*$^{-/-}$ F, n= 5; B6.NOD-*Aec1Aec2.Il17*$^{-/-}$ M, n= 5). Red arrows indicate macrophages and black arrows indicate neutrophils. Images are representatives at 400X magnification

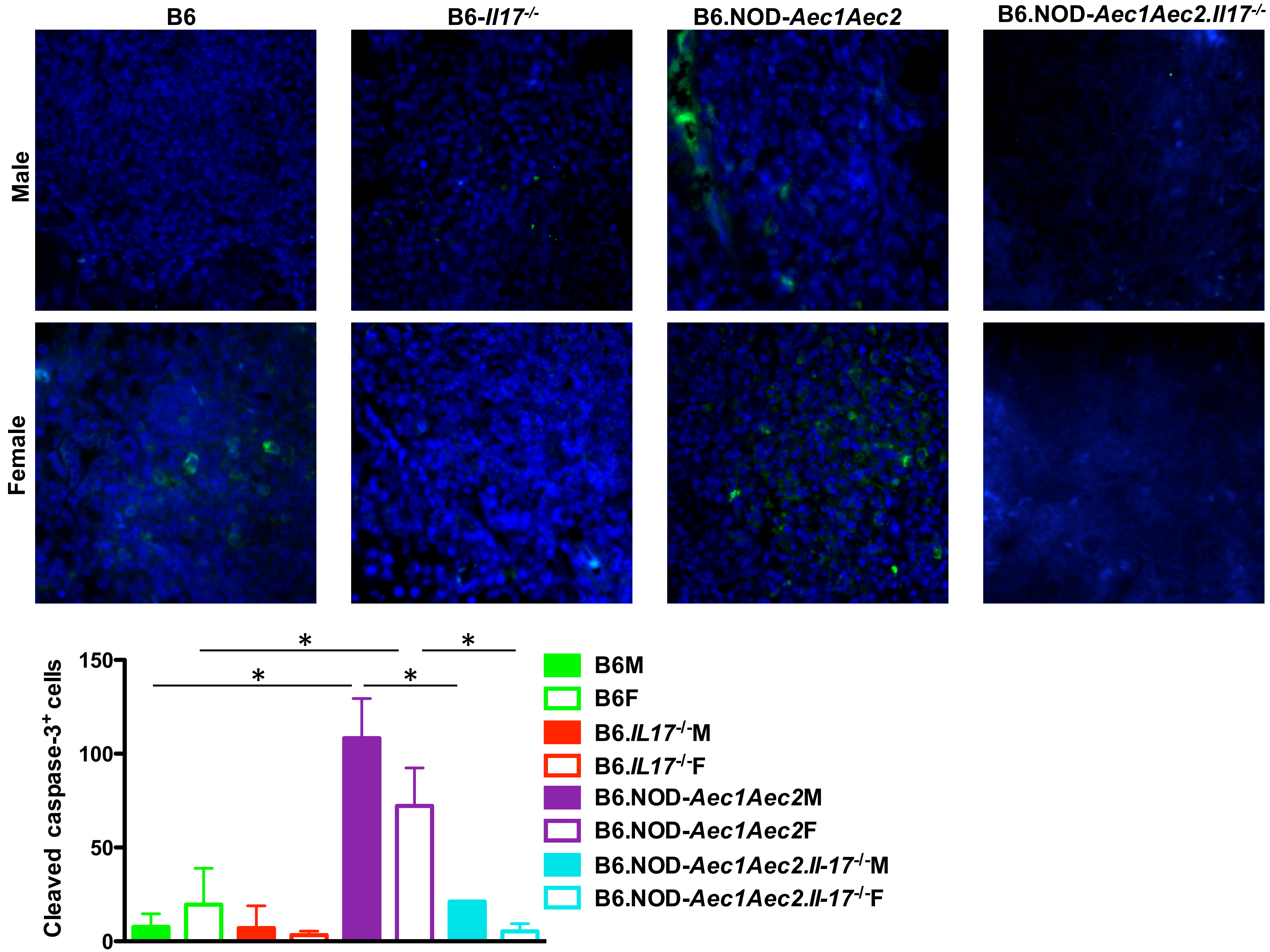
B6
B6-Il17-/-
B6.NOD-Aec1Aec2
B6.NOD-Aec1Aec2.Il17-/-
Male
Female
Cleaved caspase-3+ cells
150
100
50
0
*
*
*
*
B6M
B6F
B6.IL17-/-M
B6.IL17-/-F
B6.NOD-Aec1Aec2M
B6.NOD-Aec1Aec2F
B6.NOD-Aec1Aec2.Il-17-/-M
B6.NOD-Aec1Aec2.Il-17-/-F

Supplementary Fig. S2. Caspase-3 apoptotic signaling in the salivary glands*.*

Paraffin-embedded tissues of the salivary glands were sectioned and mounted onto microscope slides. Slides were pressure-cooked in Trilogy (Cell Marque, Rocklin, CA) according to manufacturer's instructions to deparaffinize and dehydrate slides. Slides were blocked for an hour with donkey serum, followed by overnight incubation with anti-mouse caspase3 (ThermoFisher, Waltham, MA). Secondary antibody treatment consisted of 1:100 AF488 (Invitrogen, Carlsbad, CA). Stained sections were mounted using Vectashield DAPI-mounting medium and visualized at 200X magnification using NikonTi-E fluorescent microscope.

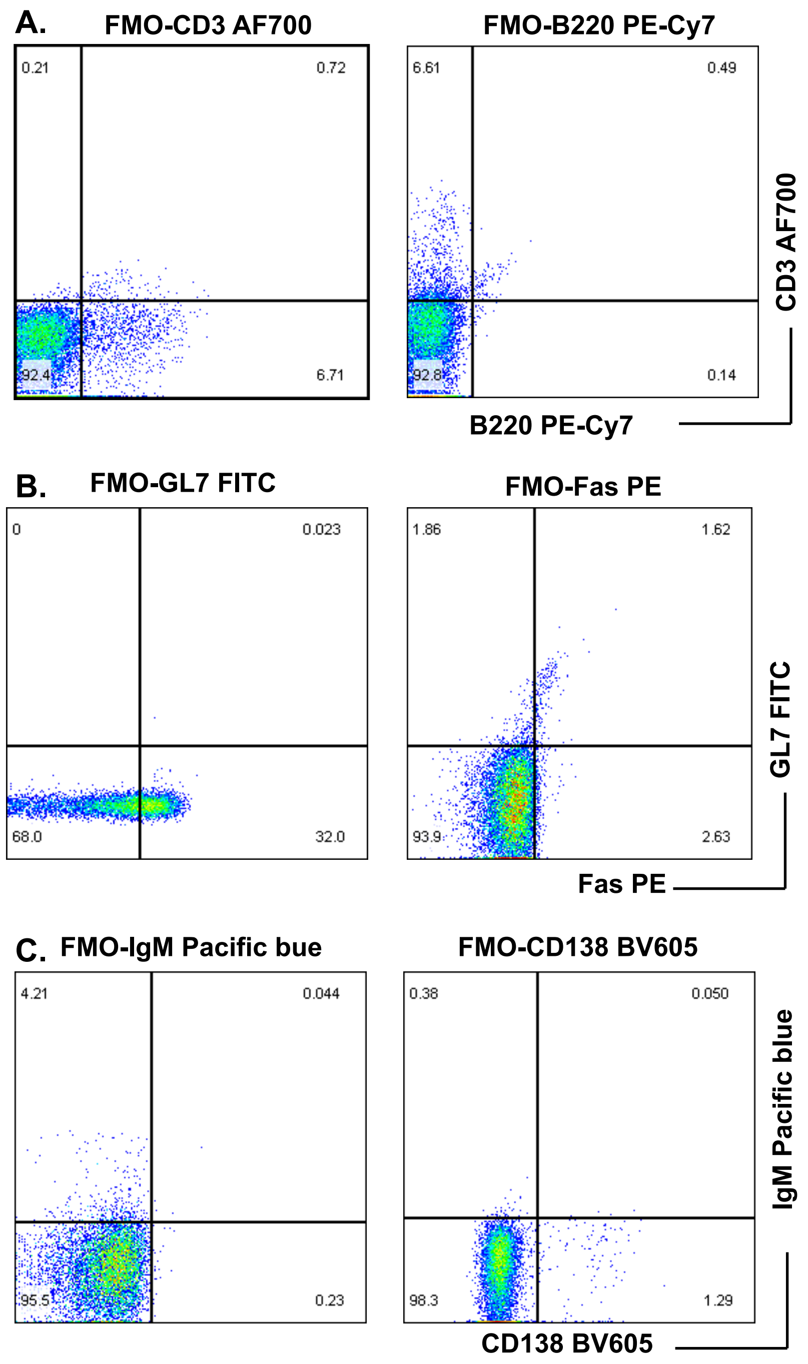
A.
FMO-CD3 AF700
0.21
0.72
92.4
6.71
FMO-B220 PE-Cy7
6.61
0.49
92.8
0.14
CD3 AF700
B220 PE-Cy7
B.
FMO-GL7 FITC
0
0.023
68.0
32.0
FMO-Fas PE
1.86
1.62
93.9
2.63
GL7 FITC
Fas PE
C.
FMO-IgM Pacific bue
4.21
0.044
95.5
0.23
FMO-CD138 BV605
0.38
0.050
98.3
1.29
IgM Pacific blue
CD138 BV605

Supplementary Fig. S3. Fluorescence minus one (FMO) controls. Cells were stained with all fluorochrome-conjugated antibodies in the experimental samples except the one of interest (FMO-anti-CD3 AF700, anti-B220 PE-Cy7, anti-GL7 FITC, anti-Fas PE, anti-IgM Pacific blue, anti-CD138 BV605). Displayed are the images of the comparative parameters shown elsewhere in the study. **A**. Anti-CD3 AF700 versus anti-B220 PE-Cy7, **B**. Anti-GL7 FITC versus anti-Fas PE, and **C**. Anti-IgM Pacific blue versus anti-CD138 BV605.
